# Is red cell distribution width a prognostic factor in patients with breast cancer? A meta-analysis

**DOI:** 10.3389/fsurg.2023.1000522

**Published:** 2023-03-24

**Authors:** Jun-Ming Yin, Ke-Peng Zhu, Zhi-Wei Guo, Wen Yi, Ying He, Guo-Cheng Du

**Affiliations:** Mastothyroid Vascular Surgery, The Second Clinical Medical College of North Sichuan Medical College Nanchong Central Hospital, Nanchong, China

**Keywords:** breast cancer, red blood cell distribution width, overall survival, disease-free survival, meta-analysis

## Abstract

**Purpose:**

The current study aimed to investigate whether red blood cell distribution width (RDW) can predict the prognosis of patients with breast cancer (BC).

**Methods:**

We searched four databases, including PubMed, Embase, Cochrane Library databases, and CNKI, from inception to Jun 13, 2022. The primary outcome was overall survival (OS), and the secondary outcome was disease-free survival (DFS). A subgroup analysis was conducted based on different treatments. This meta-analysis was performed with RevMan 5.3 (The Cochrane Collaboration, London, United Kingdom).

**Results:**

A total of seven studies including 4,884 BC patients were identified. The high RDW group had a larger tumor size (OR = 2.12, 95% CI = 1.67 to 2.68, *P* < 0.01), higher proportions of advanced stage tumors (OR = 1.77, 95% CI = 1.38 to 2.27, *P* < 0.01), more lymph node metastases (OR = 2.00, 95% CI = 1.58 to 2.51, *P* < 0.01) and lower HER-2 expression (OR = 0.76, 95% CI = 0.61 to 0.95, *P* = 0.02). For prognosis, after pooling all the data, we found that the high RDW group was associated with worse OS (HR = 2.12, 95% CI = 1.47 to 3.08, *P* < 0.01) and DFS (HR = 1.77, 95% CI = 1.32 to 2.37, *P* < 0.01). The subgroup analysis found that RDW had prognostic significance but only for surgery-only patients (HR = 2.41, 95% CI = 1.67 to 3.49, *P* < 0.01).

**Conclusion:**

High RDW was associated with worse OS and DFS. Therefore, RDW was a simple predictive factor for the prognosis of BC patients.

## Introduction

Breast cancer (BC) is one of the most common cancers and the second leading cause of cancer-related death in women worldwide (1, 2). Approximately 1.5 million women are diagnosed with BC each year, and this number is expected to increase to 2.2 million annually by 2025 (3). There are different treatments, including systemic therapy, surgery, and radiotherapy, depending on the stage of BC (4–6). Therefore, convenient preoperative predictive values for BC prognosis could help surgeons develop treatment strategies and improve surgical outcomes.

Red blood cell distribution width (RDW) is a simple and readily available parameter that represents the heterogeneity of red blood cell volume and is traditionally used in the differential diagnosis of anemia (7). Elevated RDW can predict mortality and morbidity in patients with benign diseases, including cerebral infarction (8), acute myocardial infarction (9), pancreatitis (10), pulmonary embolism (11), acute renal failure (12), coronary artery disease, and heart failure (13, 14). It is also a marker for predicting the prognosis of tumors such as gastric cancer (15), esophageal cancer (16), hepatocellular carcinoma, and colorectal cancer (17, 18).

However, for BC, the effect of RDW on prognosis is controversial (19–25). Wang C et al. analyzed 443 BC patients and found that RDW was not a prognostic factor for OS (19). Similarly, Takeuchi H et al. analyzed 299 BC patients and found that RDW was not a predictor for DFS (20). However, Yoo YC et al. demonstrated that high RDW had high predictive power for OS and DFS (21). In another study reported by Yao D et al. high pretreatment RDW levels in BC patients were associated with poor OS and DFS; thus, RDW could be a potential predictive factor in determining poor prognosis in all from all patients (23). Therefore, it is necessary to identify the exact role of RDW in the prognosis of BC patients.

## Materials and methods

This meta-analysis was conducted following the Preferred Reporting Items for Systematic Reviews and Meta-Analyses (PRISMA) statement (26).

### Literature search strategy

We searched four databases, including PubMed, Embase, Cochrane Library databases, and CNKI, from inception to Jun 13, 2022. The search strategy included two keywords: RDW and BC. For RDW, the search strategy was as follows: “red blood cell distribution width” OR “red cell distribution width” OR “RDW”. In terms of BC, the search strategy was as follows: “Breast Neoplasms” OR “Breast Cancer” OR “Breast Tumor” OR “Breast Tumors” OR “Breast Carcinoma” OR “Breast Carcinomas”. Then, we use “AND” to combine the two keywords. The languages were limited to English and Chinese.

### Inclusion and exclusion criteria

Our meta-analysis aimed to analyze the effect of RDW on the prognosis of BC, therefore, the inclusion criteria for studies were as follows: (1) the patients included were diagnosed with primary BC; (2) the study included both a control group (the low RDW group) and an exercise group (the high RDW group); (3) the study reported the prognosis including overall survival (OS) or disease-free survival (DFS); and (4) the study was published in English or Chinese. The exclusion criteria for studies were as follows: (1) the article type was a case report, a review, a letter to the editor, comments, or conference literature; and (2) there was an absence of the full text. Two reviewers conducted the inclusion and exclusion criteria, separately. Disagreement was settled by group discussion.

### Study selection

Two reviewers searched the four databases. The duplicated studies were eliminated first. Then, the titles and abstracts were screened to find eligible studies. After that, the full texts were checked to determine whether the studies were suitable for the final analysis. Two reviewers conducted the study selection, and the final judgment was made after a group discussion.

### Data extraction

The data included the study information, baseline information, and prognostic information. The study information included the first author, publishing year, publishing country, and Newcastle-Ottawa Scale (NOS) score. The baseline information included the study data, patient information, sample size, and cutoff value of RDW. The prognostic information included OS and DFS. These data were extracted independently and cross-checked by two reviewers.

### Definitions and outcomes

OS was defined as the time from diagnosis to death due to any cause. DFS was defined as the time from diagnosis to the time of recurrence, death, or last follow-up. The primary outcome was OS, and the secondary outcome was DFS.

### Quality assessment

The NOS score was used to evaluate the quality of the included studies (27). A score of nine points represented high quality; a score of seven to eight points represented medium quality; and low-quality studies scored less than seven points.

### Statistical analysis

In the current meta-analysis, dichotomous variables including tumor diameter, tumor stage, type of surgery, chemotherapy, lymph node metastases, peritumoral vascular invasion, and estrogen receptor (ER)/progesterone receptor (PR) positivity, were collected, and odds ratios (ORs) plus 95% confidence intervals (CIs) were calculated. For OS and DFS, hazard ratios (HRs) plus 95% CIs were calculated. A subgroup analysis was conducted according to the different treatments for patients. The *I*^2^ value and the chi-squared test were used to assess the statistical heterogeneity (28, 29). When *I*^2 ^> 50%, the random effects model was used, and *p* < 0.1 was considered statistically significant. The fixed effects model was used when *I*^2 ^≤ 50%, and *p* < 0.05 was considered statistically significant. This meta-analysis was performed with RevMan 5.3 (The Cochrane Collaboration, London, United Kingdom).

## Results

### Study selection

**Figure 1:**
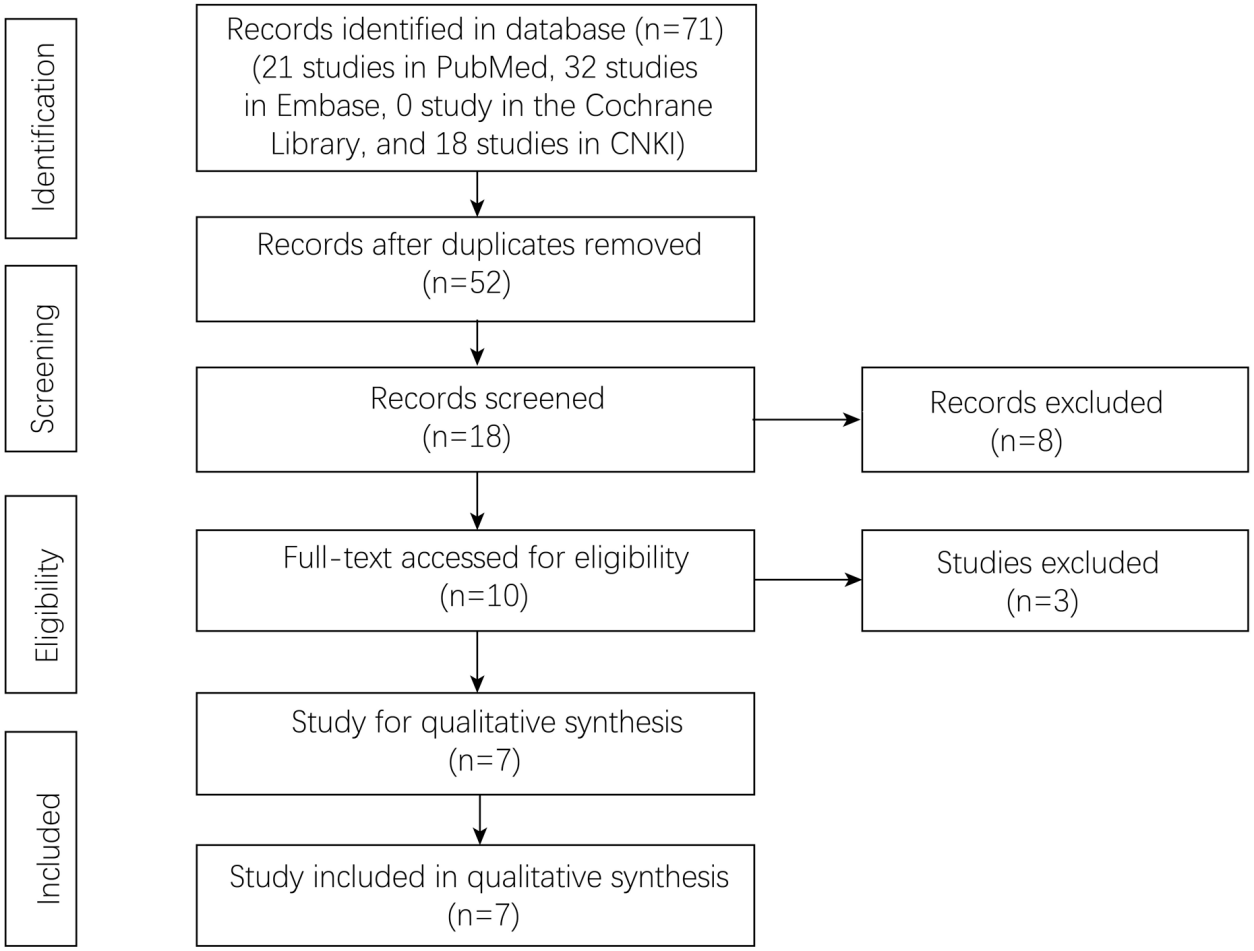
Flowchart of study selection.

A total of 71 studies were identified in the four databases (21 studies in PubMed, 32 studies in Embase, 0 studies in the Cochrane Library, and 18 studies in CNKI). There were 52 studies after removing the duplicated studies. Finally, seven studies were left for the final analysis (Figure 1).

### Patient characteristics and quality assessment of the included studies

A total of seven studies including 4,884 BC patients were identified (19–25). The publishing year was from 2014 to 2021. Five studies were published in China, one study was published in Korea and one study was published in Japan. The study period was from 1996 to 2017. For the prognosis, six studies reported OS, and five studies reported DFS. The sample size, treatment, cutoff value, and NOS score of each included study are shown in Table 1.

**Table 1:** Baseline characteristics of included studies.

Author	Year	Country	Study date	Sample size	Patients	Treatment	Survival volume	Cut-off volume	NOS
Takeuchi H	2019	Japan	2006–2017	299	M0 BC	Surgery	DFS	13.7%	7
Yoo YC	2021	Korea	2005–2010	1783	Invasive BC	Surgery, neoadjuvant therapy, chemotherapy or radiotherapy	OS/DFS	13.5%	9
Li F	2018	China	2010–2012	280	Invasive M0 BC	Surgery and chemotherapy (no neoadjuvant therapy before surgery)	OS/DFS	13.45%	7
Yao D	2019	China	2009–2014	825	Invasive M0 BC	Surgery (no neoadjuvant therapy, chemotherapy or radiotherapy)	OS/DFS	13.82%	9
Huang DP	2016	China	2008–2012	203	Invasive BC under 40 years old	Surgery or chemotherapy (no neoadjuvant therapy before surgery)	OS/DFS	13.75%	7
Yao M	2014	China	2009–2011	608	BC	no neoadjuvant therapy before surgery	OS	13.45%	8
Wang C	2014	China	1996–2011	886	Primary invasive BC	Any kind of clinical treatment, such as surgery, chemotherapy, radiation therapy, or hormone therapy	OS	14.5%	9

Abbreviations: NOS, newcastle-ottawa scales; BC, breast cancer; OS, overall survival; DFS, disease-free survival.

### Baseline information

The baseline information including tumor diameter, tumor stage, type of surgery, chemotherapy, lymph node metastases, peritumoral vascular invasion, ER/PR positivity, HER-2, and Ki-67, was compared between the high RDW group and the low RDW group. The high RDW group had a larger tumor size (OR = 2.12, 95% CI = 1.67 to 2.68, *P* < 0.01), a higher proportion of advanced stage tumors (OR = 1.77, 95% CI = 1.38 to 2.27, *P* < 0.01), more lymph node metastases (OR = 2.00, 95% CI = 1.58 to 2.51, *P* < 0.01) and lower HER-2 expression (OR = 0.76, 95% CI = 0.61 to 0.95, *P* = 0.02) (Table 2).

**Table 2:** Summary of characteristics between the high RDW group and the Low RDW group.

Characteristics	Studies	Participants (the High RDW/the Low RDW)	Odds Ratio/Mean Difference (95% CI)	Model	Heterogeneity
Tumor diameter					
≤5	3	546/762	Reference	Reference	Reference
>5	3	546/762	2.12 [1.67, 2.68]; *P* < 0.00001	FE	*I*^2 ^= 3%; *P* = 0.36
TNM stage					
I	3	Reference	Reference	Reference	Reference
II	3	435/881	3.21 [0.23, 44.75]; *P* = 0.39	RE	*I*^2 ^= 99%;
III	3	546/762	1.77 [1.38, 2.27]; *P* < 0.00001	FE	*P* < 0.00001
					*I*^2 ^= 23%; *P* = 0.27
Type of surgery					
Conservation	3	546/362	Reference	Reference	Reference
Radical	3	546/352	0.81 [0.44,1.47]; *P* = 0.48	FE	*I*^2 ^= 8%; *P* = 0.30
Chemotherapy					
FEC	3	Reference	Reference	Reference	Reference
TAC/TEC	3	500/684	0.80 [0.61, 1.06]; *P* = 0.12	FE	*I*^2 ^= 29%; *P* = 0.12
Non	3	546/762	0.79 [0.54, 1.17]; *P* = 0.25	FE	*I*^2 ^= 0%; *P* = 0.25
Lymph node metastases	3	546/762	2.00 [1.58, 2.51]; *P* < 0.00001	FE	*I*^2 ^= 0%; *P* = 0.83
peritumoral vascular invasion	2	454/574	1.07 [0.45, 2.50]; *P* = 0.88	RE	*I*^2 ^= 60%; *P* = 0.12
ER positive	4	756/1160	1.03 [0.85, 1.25]; *P* = 0.76	FE	*I*^2 ^= 0%; *P* = 0.72
PR positive	4	756/1160	1.13 [0.94, 1.37]; *P* = 0.19	FE	*I*^2 ^= 0%; *P* = 0.80
HER-2	4	756/1160	0.76 [0.61, 0.95]; *P* = 0.02	FE	*I*^2 ^= 0%; *P* = 0.82
Ki-67	3	546/762	1.04 [0.83, 1.31]; *P* = 0.72	FE	*I*^2 ^= 0%; *P* = 1.00

RDW, red blood cell distribution width; CI, confidence intervals.

### OS

**Figure 2:**
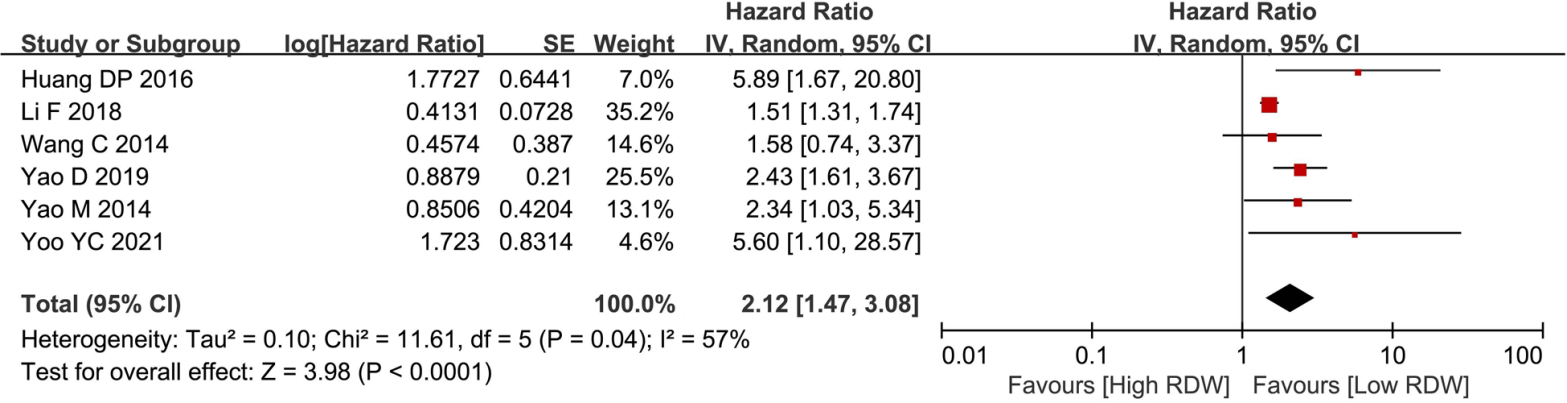
Os between the high RDW group and the low RDW group. Abbreviation: OS, overall survival; RDW, red blood cell distribution width.

Six studies with 4,585 patients reported OS data on BC patients. After pooling all the data, we found that the high RDW group was associated with worse OS than the low RDW group (HR = 2.12, 95% CI = 1.47 to 3.08, *P* < 0.01) (Figure 2).

### DFS

**Figure 3:**
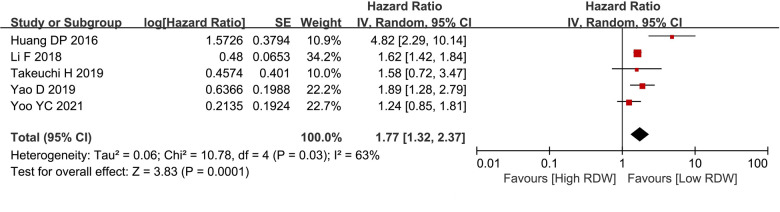
DFS between the high RDW group and the low RDW group. Abbreviation: DFS, disease-free survival; RDW, red blood cell distribution width.

Five studies with 3,390 patients reported data on DFS in BC patients. After pooling all the data, we found that the high RDW group was associated with worse DFS than the low RDW group (HR = 1.77, 95% CI = 1.32 to 2.37, *P* < 0.01) (Figure 3).

### Subgroup analysis for Os

**Figure 4:**
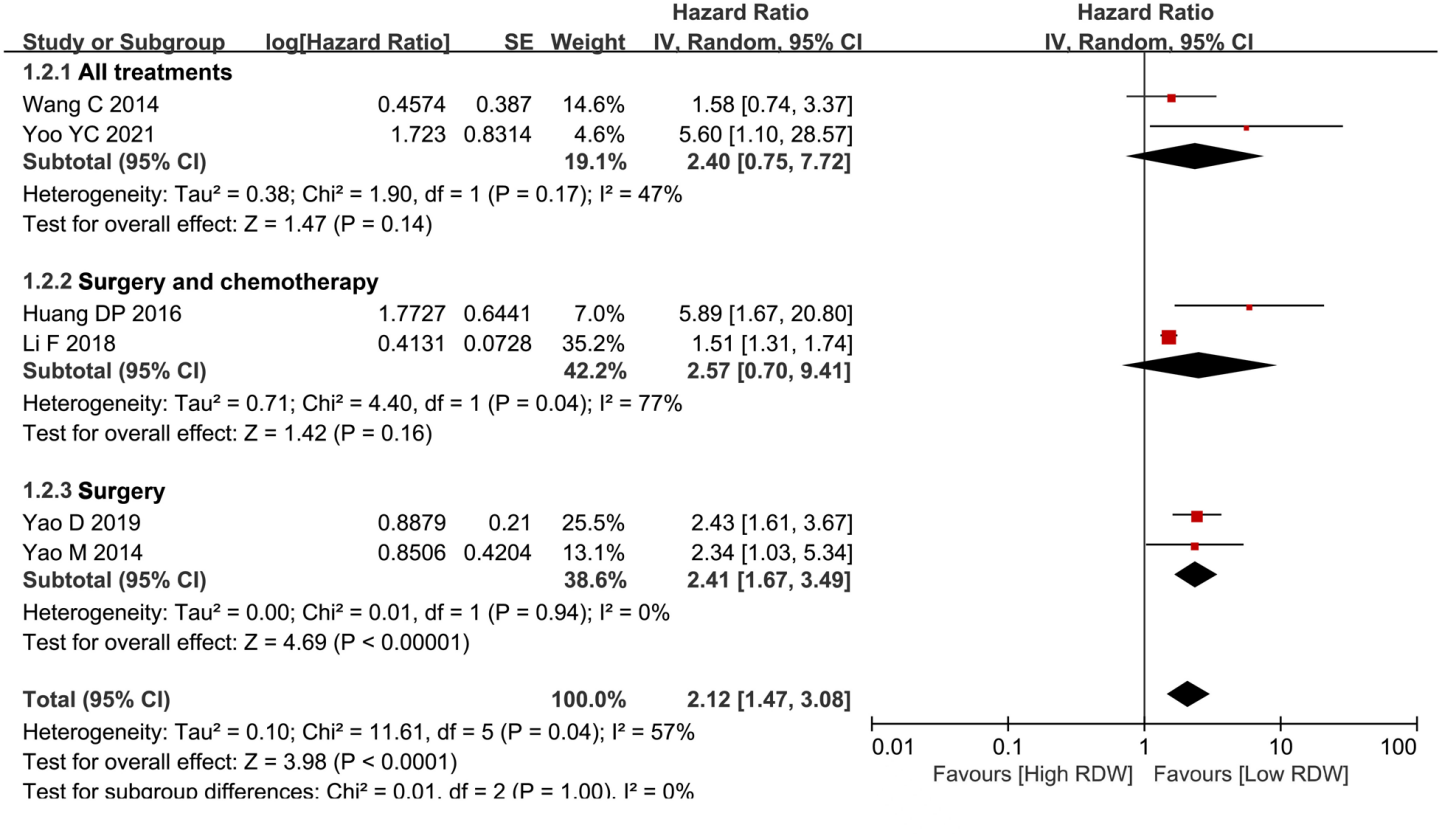
Subgroup analysis for OS based on treatment. Abbreviation: RDW, red blood cell distribution width.

According to the different treatments, the BC patients were divided into three groups. Two studies included patients who received surgery, neoadjuvant treatment or adjuvant treatment, two studies included patients who received surgery or adjuvant treatment, and two studies included patients who only underwent surgery. After subgroup analysis, RDW had prognostic significance only for the surgery-only patients (HR = 2.41, 95% CI = 1.67 to 3.49, *P* < 0.01) but not for the all-treatment groups (HR = 2.40, 95% CI = 0.75 to 7.72, *P* = 0.14) and the neoadjuvant treatment groups (HR = 2.57, 95% CI = 0.70 to 9.41, *P* = 0.16) (Figure 4).

### Sensitivity analysis

A sensitivity analysis was conducted by excluding one study at a time to examine its impact on the result. In the current meta-analysis, the sensitivity analysis was performed based on the outcomes of OS and DFS, and the subgroup analyses of OS. After each study was successively removed, the omission of any of the studies did not change the conclusion. This suggested that the outcomes had a low level of sensitivity and produced reliable results.

## Discussion

A total of seven studies including 4,884 BC patients were included in the current meta-analysis. For prognosis, after pooling all the data, we found that the high RDW group was associated with worse OS and DFS than the low RDW group, especially for BC patients who underwent only radical surgery. Therefore, we concluded that RDW could be widely used in the clinic as an easy preoperative prognostic predictor. Surgeons should pay more attention to patients with high preoperative RDW levels and take action in advance to prolong the survival time of BC patients.

Although many new prognostic markers have been explored and identified, the major problem with these biomarkers is that they heavily rely on complex molecular or genetic tests (30–32). Hematological parameters, including albumin, C-reactive protein (CRP), neutrophils, and lymphocytes, are readily available and inexpensive parameters for BC patients that could predict the prognosis (33–35). As a routinely available marker of the systemic inflammatory response, RDW predicts negative clinical outcomes in various tumors. However, there is a controversy regarding whether RDW has an impact on BC (19–25).

Of the seven included studies, two reported that RDW was a prognostic indicator (19, 20), but the other five studies reported that RDW did not affect BC (21–25). Therefore, the current study aims to investigate whether RDW can predict the prognosis of BC. If RDW could be used as an easy prognostic indicator, it would be a convenient clinical reference value. To our knowledge, our study is the first to pool all the prognostic data of RDW in BC. In our study, we found that high RDW was associated with worse OS and DFS than low RDW, which indicated that RDW was an important biomarker for BC.

The mechanisms for the relationship between RDW and poor prognosis remain complex and unclear. However, some hypotheses accounted for the mechanisms. One hypothesis was that oxidative stress (23, 36) might reduce the survival of red blood cells and lead to elevated RDW. Both endogenous and exogenous sources of reactive oxygen species (ROS) can lead to increased oxidative stress in cells (37). Moreover, excessive ROS can cause damage and modification of cellular macromolecules, thus mutating genomic DNA. Another hypothesis was that chronic inflammation (38) could induce an increase in RDW by disrupting the erythrocyte membrane, leading to changes in erythrocyte maturation. Inflammation in the microenvironment could promote tumor growth, invasion, angiogenesis, and ultimately metastasis of BC (39, 40). This was corroborated by our finding that patients with high RDW had larger tumor sizes, more advanced tumor stages, and were more likely to have lymph node metastases.

Thus, for clinicians, it is critical to pay more attention to monitoring patients with high preoperative RDW. Minimizing RDW before surgery and providing interventions such as nutritional support or anti-inflammatory drugs are necessary treatment strategies (21, 41).

There were some limitations in our meta-analysis. First, the seven included studies were relatively small with a small number of BC patients, which might cause bias; Second, the cut-off of RDW was inconsistent, which might cause heterogeneity; Third, all the included studies were from Asia, the lack of other regions might also lead to selection bias. Therefore, multicenter, multiregional, prospective, and high-quality RCTs should be carried out in the future.

In conclusion, high RDW was associated with worse OS and DFS. Therefore, RDW was a simple predictive factor for the prognosis of BC patients.

## Data Availability

The raw data supporting the conclusions of this article will be made available by the authors, without undue reservation.
