# Supplementary material for: Is red cell distribution width a prognostic factor in patients with breast cancer? A meta-analysis
Source: Front Surg. 2023 Mar 24;10:1000522. doi: 10.3389/fsurg.2023.1000522 (PMC10079877; doi:10.3389/fsurg.2023.1000522)
Supplement: Supplementary file 1 [file Table1.docx]

PubMed

| Search number | Query |
| --- | --- |
| #1 | (red blood cell distribution width[Title/Abstract]) OR (red cell distribution width[Title/Abstract]) OR (RDW[Title/Abstract]) |
| #2 | (Breast Neoplasms[Title/Abstract]) OR (Breast Cancer[Title/Abstract]) OR (Breast Tumor[Title/Abstract]) OR (Breast Tumors[Title/Abstract]) OR (Breast Carcinoma[Title/Abstract]) OR (Breast Carcinomas[Title/Abstract]) |
| #3 | #1 AND #2 |

Embase

| Search number | Query |
| --- | --- |
| #1 | 'red blood cell distribution width':ti,ab,kw OR 'red cell distribution width':ti,ab,kw OR 'rdw':ti,ab,kw |
| #2 | 'breast neoplasms':ti,ab,kw OR 'breast cancer':ti,ab,kw OR 'breast tumor':ti,ab,kw OR 'breast tumors':ti,ab,kw OR 'breast carcinoma':ti,ab,kw OR 'breast carcinomas':ti,ab,kw |
| #3 | #1 AND #2 |

Cochrane Library

| Search number | Query |
| --- | --- |
| #1 | (red blood cell distribution width OR red cell distribution width OR RDW):ti,ab,kw |
| #2 | (breast neoplasms OR breast cancer OR breast tumor OR breast tumor OR breast carcinoma OR breast carcinomas):ti,ab,kw |
| #3 | #1 AND #2 |

CNKI

| Search number | Query |
| --- | --- |
| #1 | 红细胞分布宽度[篇关摘] |
| #2 | 乳腺癌[篇关摘] |
| #3 | #1 AND #2 |
